# Effects of Recent Minimum Temperature and Water Deficit Increases on *Pinus pinaster* Radial Growth and Wood Density in Southern Portugal

**DOI:** 10.3389/fpls.2016.01170

**Published:** 2016-08-12

**Authors:** Cathy B. Kurz-Besson, José L. Lousada, Maria J. Gaspar, Isabel E. Correia, Teresa S. David, Pedro M. M. Soares, Rita M. Cardoso, Ana Russo, Filipa Varino, Catherine Mériaux, Ricardo M. Trigo, Célia M. Gouveia

**Affiliations:** ^1^Instituto Dom Luiz, Faculdade de Ciências, Universidade de LisboaLisboa, Portugal; ^2^Centro de Investigação e de Tecnologias Agro-Ambientais e Biológicas, Universidade de Trás-os-Montes e Alto DouroVila Real, Portugal; ^3^Biosystems and Integrative Sciences Institute, Universidade de Lisboa Faculdade de CiênciasLisboa, Portugal; ^4^Centro de Estudos Florestais, Instituto Superior de Agronomia, Universidade de LisboaLisboa, Portugal; ^5^Departamento de Genética e Biotecnologia, Universidade de Trás-os-Montes e Alto DouroVila Real, Portugal; ^6^Instituto Nacional de Investigação Agraria e VeterináriaOeiras, Portugal; ^7^Centre National de Recherches Météorologiques, Meteo-France/CNRSToulouse, France; ^8^School of Physics and Astronomy, Monash University, ClaytonVIC, Australia

**Keywords:** maritime pine, climate change, wood radial density, wood radial growth, dendrochronology, SPEI drought index, IADFs

## Abstract

Western Iberia has recently shown increasing frequency of drought conditions coupled with heatwave events, leading to exacerbated limiting climatic conditions for plant growth. It is not clear to what extent wood growth and density of agroforestry species have suffered from such changes or recent extreme climate events. To address this question, tree-ring width and density chronologies were built for a *Pinus pinaster* stand in southern Portugal and correlated with climate variables, including the minimum, mean and maximum temperatures and the number of cold days. Monthly and maximum daily precipitations were also analyzed as well as dry spells. The drought effect was assessed using the standardized precipitation-evapotranspiration (SPEI) multi-scalar drought index, between 1 to 24-months. The climate-growth/density relationships were evaluated for the period 1958-2011. We show that both wood radial growth and density highly benefit from the strong decay of cold days and the increase of minimum temperature. Yet the benefits are hindered by long-term water deficit, which results in different levels of impact on wood radial growth and density. Despite of the intensification of long-term water deficit, tree-ring width appears to benefit from the minimum temperature increase, whereas the effects of long-term droughts significantly prevail on tree-ring density. Our results further highlight the dependency of the species on deep water sources after the juvenile stage. The impact of climate changes on long-term droughts and their repercussion on the shallow groundwater table and *P. pinaster*’s vulnerability are also discussed. This work provides relevant information for forest management in the semi-arid area of the Alentejo region of Portugal. It should ease the elaboration of mitigation strategies to assure *P. pinaster*’s production capacity and quality in response to more arid conditions in the near future in the region.

## Introduction

Trees are continuously responding physiologically to the prevailing climatic conditions. Climate and environmental factors are well known to affect wood formation at different scales, from the cell to the entire tree ring (Fritts, 1976). These factors have an impact on the amount and characteristics of the xylem cells, as well as the properties of wood rings and their density (Fritts, 1976; Vieira et al., 2014). For temperate woody species the combination of temperature and water availability mostly controls wood radial increment (Carrer and Urbinati, 2006). In drier regions such as the Mediterranean, it is, however, often assumed that tree growth is mostly limited by water availability (Cherubini et al., 2003; Gouveia et al., 2008). For example, Lebourgeois et al. (2012) succeeded in linking narrower conifer trees to years with drier springs. Also positive effects of spring precipitation changes on tree cambial activity have been reported for several Mediterranean ligneous species, such as the stone pine (*Pinus pinea* L.), the Aleppo pine (*P. halepensis* Mill.) and the maritime pine (*P. pinaster* Ait.) (Vieira et al., 2010; Campelo et al., 2013; De Luis et al., 2013). Nevertheless, Correia et al. (2008) found that water use efficiency (WUE) assessed by the variation of δ^13^C, was better explained by the mean minimum temperatures of the coldest month than by annual precipitation for a Maritime pine provenance test in Portugal. *P. pinaster* trees from contrasting altitudes presenting significant variations of the phenotypic traits at a mature stage also differed in their cone, seed and germination traits, which could be explained by a response to different minimum temperatures at the provenance origin (Correia et al., 2010, 2014).

Most of the studies evaluating tree response to climate changes are focusing on tree rings width and wood growth. Yet wood density is a highly relevant property of wood quality traits. It provides an excellent indicator of strength and yield. It is also related to the optimization of wood conversion processes (*cutting, gluing, finishing, drying, and paper making*) (Barnett, 2003). Besides wood density is also useful to assess carbon stored in tree stems, which is essential for the estimation of the carbon footprint of the forestry sector (Ilic et al., 2000). Wood density fluctuates intra-annually from less dense earlywood to denser latewood (Fritts, 1976). It can also vary at a lower scale within earlywood or latewood due to the development of latewood-like cells within earlywood or earlywood-like cells within latewood (Fritts, 2001). Those variations are called IADFs and are mostly the result of tree genetics and aging and/or responses to environmental conditions, including climate, soil, and rooting depth (Campelo et al., 2007, 2013; Novak et al., 2013; Nabais et al., 2014; Battipaglia et al., 2016; De Micco et al., 2016). The variation in density in hardwoods is to some extent linked to wood growth rate as well as wood fibers and vessel dimensions. In the case of gymnosperm softwoods and the pine gender *Pinus* sp in particular, lower densities are a result of a higher proportion of tracheids with a larger extend of cavities where the amount of cell wall material is lower. This results in lower wood density (Tsoumis, 1991; Haygreen and Bowyer, 1996; Hoadley, 2000). In addition, significant correlations were found between wood density, tracheid radial diameter and climate variables such as temperature and standardized precipitation-evapotranspiration (SPEI) drought index for the endemic long-lived conifer Huon pine in Australia (Drew et al., 2013). In the Mediterranean area, Pasho et al. (2012) observed that Aleppo pine trees presented a bigger proportion of latewood tracheids in response to decreased precipitation and high temperatures. In a dry inner Alpine valley, however, an irrigation experiment revealed that non irrigated trees presented tracheids with a wider lumen than control trees (Eilmann et al., 2011). In general, xylem anatomy response to climate changes appears to change considerably between environments and/or species (Fonti and Jansen, 2012).

In Europe, maritime pine is widely spread over the Mediterranean basin, particularly in France, Iberian Peninsula, Italy and North Africa (Carrión et al., 2000). In Portugal, the species covers about 23% of the national forested area, (ICNF, 2013). The forest species contributes notably to the Portuguese economy representing roughly 11% of the total value of forest product exports (Aguiar et al., 2003; Correia et al., 2004), as it is mostly used for carpentry, construction, chipboard, pulp, and paper production, floor boards and palettes, and high quality resin (Oppen and Hone, 1995; Alía and Martín, 2003; Gaspar et al., 2009). This species can cope with a large range of climate, altitude and soil types. Nevertheless, the consequences of recent climate changes on tree-ring traits have not been fully assessed.

Climate models consistently predict marked increases in temperature associated to decreases in precipitation in the Mediterranean basin (Diffenbaugh and Giorgi, 2012; Lionello, 2012). The occurrence of extreme events, including extreme precipitation or extreme lack of precipitation associated to high temperature is also expected to increase, leading to high-impact droughts, and heatwaves (Kundzewicz et al., 2006; Lionello, 2012; IPCC, 2013). The increase of drought incidences and intensities together with a higher variability of hydrological cycles are thus expected to affect water-supply patterns and reduce stored water availability. Such remarkable changes will prejudice forests and their related ecological, economical, sociocultural and landscape services (Schröter et al., 2005), especially in the Mediterranean basin amplifying significantly the fire risk (Sousa et al., 2015).

A significant change of the precipitation in the Iberian Peninsula has especially been characterized by a noticeable higher intra and inter-annual variability over the last three decades (García-Barrón et al., 2011; Santo et al., 2014b). According to Fragoso and Gomes (2008) the Alentejo region in southern Portugal has increasingly suffered from severe drought conditions during summer with significant drying trends observed in spring (Santo et al., 2014b). Since the 1940s, Santo et al. (2014a) observed in mainland Portugal a significant increase in the frequency and duration of heat waves, as well as an increase in the frequency of hot days especially in spring and summer. This explains why Alentejo is drifting toward an arid climate (Nunes and Seixas, 2011) expressed by a fast increase of aridity indexes since the recent past, which has highlighted a tendency toward drier climatic conditions in this region (Costa and Soares, 2012; de Figueiredo, 2013).

The objectives of this study were (1) to characterize recent climate changes in the Alentejo, a semi-arid region of Portugal, focusing on agronomical relevant climate variables and drought indexes and (2) to assess the effect of such changes on wood tree-ring width and density in *P. pinaster* trees. We report the results of over 32000 Climate-Wood relationships and analyze the impact of the recent climate variability on dendrochronological traits of *P. pinaster* in southern Portugal between 1958 and 2011.

We used an innovative approach based on the work by DeSoto et al. (2014). The temporal evolution of climate versus monthly growth and density relationships were assessed by calculating correlations framed by a moving 15-y window. The results presented as contour plots offer a 24-month window screening of the evolution of climate-growth and climate-density relationships from 1958 to 2011. Besides temperature and precipitation, further derived relevant agronomical climatic factors were also appraised. The effect of drought was assessed using the standardized precipitation-evapotranspiration index (SPEI) with temporal scales from 1 to 24 Months.

## Materials and Methods

### Sampling Location

**FIGURE 1:**
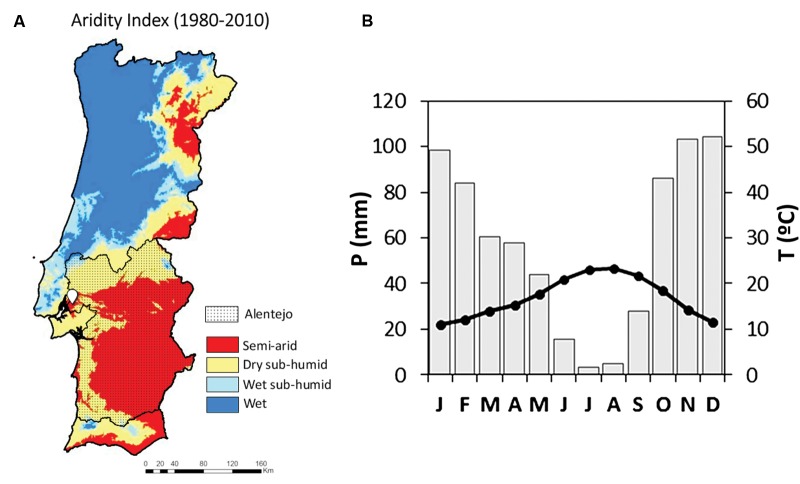
**(A)** Location of the Companhia das Lezirias sampling area (white icon) in the semi-arid Alentejo region in Portugal (dotted area). (The map of aridity index in A was adapted from PFNCNUCD (2011), courtesy of Lúcio de Rosário). **(B)** Climatic diagram of the closest ECAD grid point (located at 9.7 km from the sampling area, considering the average monthly values of precipitation and temperature of the period 1957-2012.

Wood ring cores were sampled in the northern-east part of the Alentejo region (**Figure 1A**). The sampling area was located in Companhia das Lezirias (38° 47′ 24.01 N; 8° 54′ 11.10 W) at 10-20 m above the sea level and on a gentle 1.6% slope. Long-term mean annual rainfall in the sampling location was 683 mm with a mean annual temperature of 16.07°C and 831 mm mean annual PET, using the Thornsthwaite approach (E-Obs ECA&D, 1950-2012). The Alentejo region is typically spread over a raised plain from the center to the south of Portugal. It is representative of the typical Mediterranean climate characterized by hot and dry summers and wet and cold winters as described in Köppen’s classification. Rainfall occurs predominantly from October to April, while summer water deficit prevails from June to September (**Figure 1B**). This geographic area of Portugal is considered a semi-arid region (**Figure 1A**) with a dry sub-humid climate (Nunes and Seixas, 2011). Temperature presents minimum values in January and maximum in August. In this region, the monthly mean maximum temperatures have an intra-annual range of 12 to 18°C in January and 25 to 36°C in August; whilst the minimum monthly mean temperature in January fluctuates between 3 to 9°C and 13 to 19°C in August (Soares et al., 2012). The maximum monthly daily precipitation mean occurs in December with circa 4 mm/day and the minimum occurs during the summer months, with almost no precipitation (Soares et al., 2012; Cardoso et al., 2013). The annual precipitation cycle shows another minimum in March (1.5 mm/day).

### Dendrochronological Datasets

This study presents a preliminary exploratory analysis, which is part of a large framework aiming at assessing *P. pinaster*’s plasticity response to contrasting climatic conditions in Portugal. Companhia das Lezirias is one of eleven selected sites in the framework. For this reason the sampling size in each location was limited to ten cores and only one core by tree.

Dendrochronological series considered here are the average of ten 60-year old maritime pine trees with a mean diameter at breast height of 36.9 cm and a mean height of 21.3 m. The material was collected at breast height (1.3 m), extracting one increment core per tree, from bark to pith. Using a twin-blade circular saw, a 2 mm-thick radial strip segment was sawn from each increment core and then conditioned at 12% moisture content. These radial samples were X-rayed perpendicularly to the transverse section, and their images scanned and analyzed by microdensitometric equipment (Joyce Loebl MK3) in order to compute the density components. The details of the method can be found in Gaspar et al. (2008).

Growth ring boundaries were identified in the radial density profiles by locating the sharp variations in density. Cross-examination was occasionally required, and this was done by visual assessment of the macroscopic anatomical features of the wood strip. The earlywood–latewood boundary was assigned for each ring by the average of the minimum and maximum density values within each ring (Gaspar et al., 2009). Thus, all density points within each ring with values higher than the defined boundary value for that ring were considered to be latewood.

Tree-ring cores were dated and synchronized against the reference of two characteristic wet years (1990 and 1998) and two characteristic dry years (1995, 2005) (**Supplementary Figure S1**). Age-related trends were removed from dendrochronological series through the application of an appropriate standardization methodology. This was achieved by fitting a negative exponential or polynomial smoothing spline functions on tree-ring width time-series and linear functions on density time-series (Cook and Peters, 1981; Cook, 1987) for each tree-ring core (see example of tree n°7 in **Supplementary Figure S2**). Standardized indices of tree-rings were obtained by dividing the original observed data of width or density by the best fitted exponential or linear function for each core. The final indices used to evaluate the impact of recent climate change on *P. pinaster* wood ring traits were calculated as the average of the 10 sampled core standardized indices. Here we considered the final indices of the total, early and latewood radial growth (TWG, EWG, LWG) and the mean radial density of the TWD covering the period 1958-2011. The final indices of both early and latewood mean radial density of (EWD, LWD) started after 1965 due to the removal of several outliers, which prevented the standardization function from being correctly fitted.

IADFs in *P. pinaster* were evaluated by the gradual transition in cell size and color and wall thickness within previously identified annual tree-ring boundaries (Nabais et al., 2014). IADFs were classified based on the radial position within the tree-ring (Vieira et al., 2014): IADFs classified as type E were identified as latewood-like cells within earlywood; IADFs with type L were identified when earlywood-like cells were observed within the latewood. L+ type IADFs were distinguished from L type when the gradient color of earlywood-like cells in latewood presented a homogeneous aspect. The identification of IADFs was made visually on synchronized dated cores using a binocular magnifyer and cross-validated against microdensitometric profiles. The relative frequency of IADFs was calculated as *f = n_i_/N*, where *n_i_* is the number of trees showing an IADF in the year *i* and *N* is the total number of trees observed the same year *i* (Nabais et al., 2014).

### Meteorological Datasets

Meteorological datasets covering the period 1950-2012 were retrieved from the public database of the Observational station data of the ECA&D European Climate Assessment & Dataset (Klein Tank et al., 2002) and the Observations gridded dataset form the EU-FP6 project ENSEMBLES (E-OBS, Haylock et al., 2008). Daily temperature and precipitation were extracted from the closest grid point 9.7 km away from the sampling location. Further climate variables and indexes were calculated from the E-OBS retrieved datasets on a monthly basis.

In order to maintain the analysis within a reasonable length we restricted the wide range of potential meteorological variables to the most interesting plant growth climate factors including the following precipitation-based monthly variables: the accumulated precipitation (P), the maximum daily precipitation (Pmax), the number of dry days (Dryd) and the maximum of consecutive dry days or dryspell (Drys).

Monthly temperature-based variables were also calculated and evaluated, such as the monthly average of daily mean temperature (T), the monthly averages of daily minimum (Tmin) and maximum temperatures (Tmax), the number of cold days when Tmin was lower than 5°C (Cold), the growing degree days (GDD), the sum of daily mean temperature since the 1st of January of the current year (HS1yr) and the sum of daily mean temperature since the 1st of January of the previous year (HS2yr).

Drought effects have been assessed through the SPEI multi-scalar drought index, which has become widely used in the last decade and is considered to be more appropriate for the Mediterranean type of climate, than the standard PDSI (Sousa et al., 2011; Vicente-Serrano et al., 2013). This index based on the difference between precipitation and PET is comparable in time and space (Hayes et al., 1999) and can be computed at different time scales, from one month to several years, to monitor droughts. Nonetheless, SPEI is a site-specific drought indicator of deviations from the average water balance. Thus it integrates the effect of temperature increase on droughts. SPEI was computed to analyze the effect of drought severity and short to long-term water balance deficits on tree growth and density preceding or co-occuring the wood ring formation.

### Data Processing and Statistics

Climatic series of precipitation and temperature were decomposed into seasonal and trend components to highlight long-term climatic patterns. This was achieved by computing the “STL” function using the default configuration in the R programming language, according to the Seasonal Trend decomposition procedure from Loess (Cleveland et al., 1990). Since the seasonal component of climatic variables was stationary, it was not removed from the climatic dataset before the computation of Pearson correlations coefficients.

The temporal evolution of the climate-growth relationship was analyzed according to DeSoto et al. (2014), by computing the Pearson correlation coefficients between the dendrochronological time-series and the monthly climatic times-series for the common 54-year period of 1958-2011. Pearson coefficients (*r*) were calculated considering a moving window of 15-year intervals. Each correlation coefficient provided for a specific year *y*_i_ on the y-axis of dendrochronology/climate figures was calculated as the correlation between dendrochronological variables observed from year *y_i-7_* to *y*_i+7_ and the climatic variable of the corresponding 15-year period. As an example, *r* values for 1970 presented on those figures represents the Pearson correlations from the relationship of tree-ring traits *versus* climate variables observed during the period 1963-1977. We considered a shorter moving window (15 years) compared with the 30-year interval of DeSoto et al. (2014) for two reasons: (1) Pearson correlations with *n* = 15 allowed the identification of clear climate response patterns; (2) a 30-year interval would have provided a much shorter time span on dendrochronology/climate figures, thus not allowing such a clear identification of the transition from juvenile to mature wood, nor the effect of the most recent and speeding up climate changes observed since the late 1980s.

A temporal framework of 24 consecutive months was considered as the x-axis of the dendrochronology/climate figures to identify the relationships between wood properties and key climatic factors from the previous to the current year of tree-ring xylogenesis. Though correlations were calculated for each climatic variable initially considered, here we only present graphs of the main common key climatic factors involved in *P. pinaster*’s wood ring growth and density.

We computed monthly SPEI with time-scales spanning from 1 to 24 months, which were related with maritime pine radial growth and density. We then calculated the correlations between SPEI and the corresponding dendrochronological time-series over the period 1965-2010 and for each time-scale (1 to 24 months). Therefore positive values of SPEI correspond to wet conditions while negative values indicate drought.

The impact of climatic variables on dendrochronological traits was ranked by scoring the total number of significant correlations (*P* < 0.05, |*r*| > 0.515) divided by the total number of correlations obtained in dendrochronology/climate figures, The results were expressed as the percentage of significant correlations obtained over a total of 912 (24 months^∗^38 years = 912) calculated correlations in each dataset.

## Results

### Dendrochronological Time-Series

**FIGURE 2:**
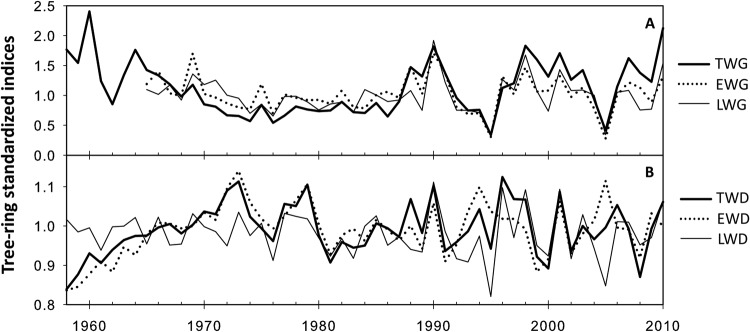
**Average of standardized time series of dendrochronological traits since 1958.** **(A)** Wood ring width. **(B)** Wood ring density.

The time-series of standardized tree-ring width and density measurements performed on *P. pinaster* cores are presented in **Figure 2**. Tree radial growth in TWG, earlywood, and LWG showed particularly high value in 1990, 1998 and 2010 while the lowest values were observed in 1995 and 2005 (**Figure 2A**). These observations, respectively, matched the characteristic wet and dry years used for core dating synchronization. Tree radial density (TWD) presented higher inter-annual variability with less obvious climatic pattern (**Figure 2B**).

### Recent Climate Evolution in Southern Portugal

**FIGURE 3:**
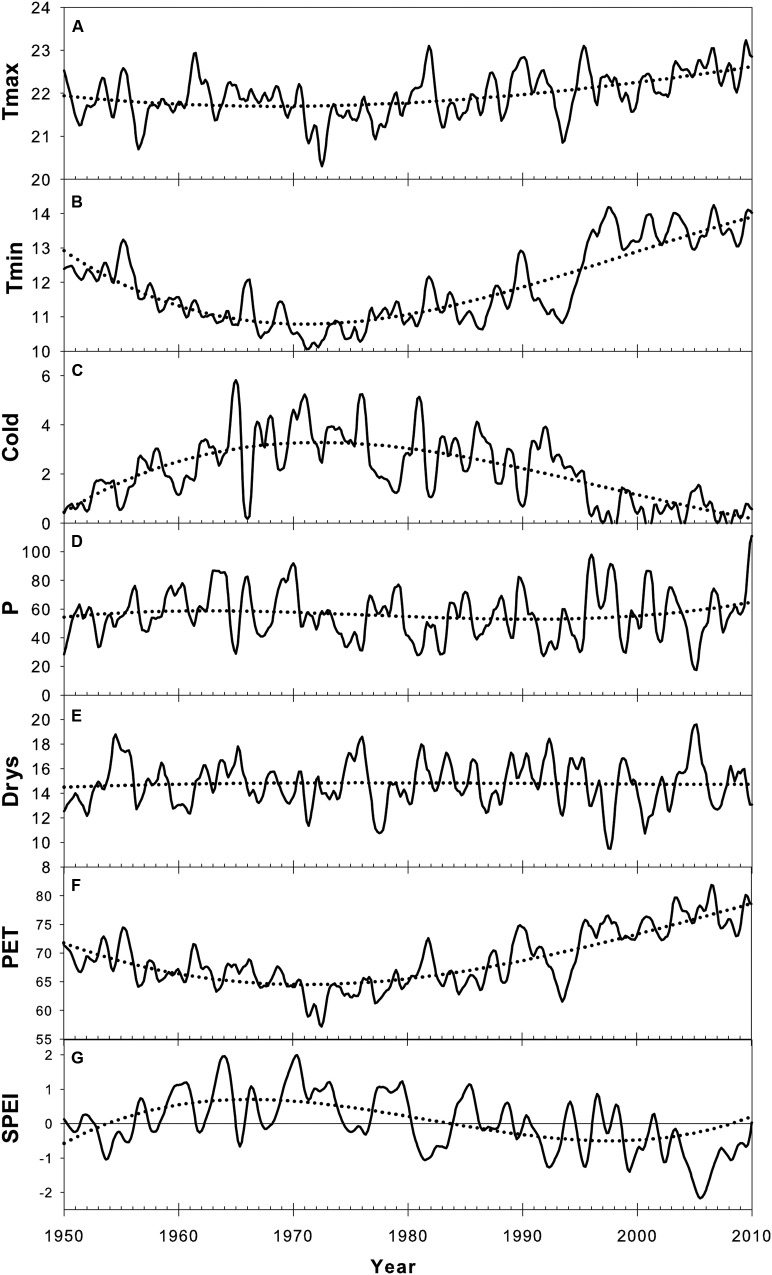
**Trend component of climate time-series according to the seasonal decomposition by Loess (STL) over the period 1950-2010.** A 3rd degree polynomial regression was fitted over STL trends to highlight multi-decadal changes (dotted curve). SPEI values are presented with a 12-month time-scale. **(A)** Maximum temperature (Tmax), **(B)** Minimum temperature (Tmin), **(C)** Number of cold days (Cold), **(D)** Precipitation (P), **(E)** Dryspell (Drys), **(F)** Potential evapotranspiration (PET), **(G)** 12-Month SPEI index.

**FIGURE 4:**
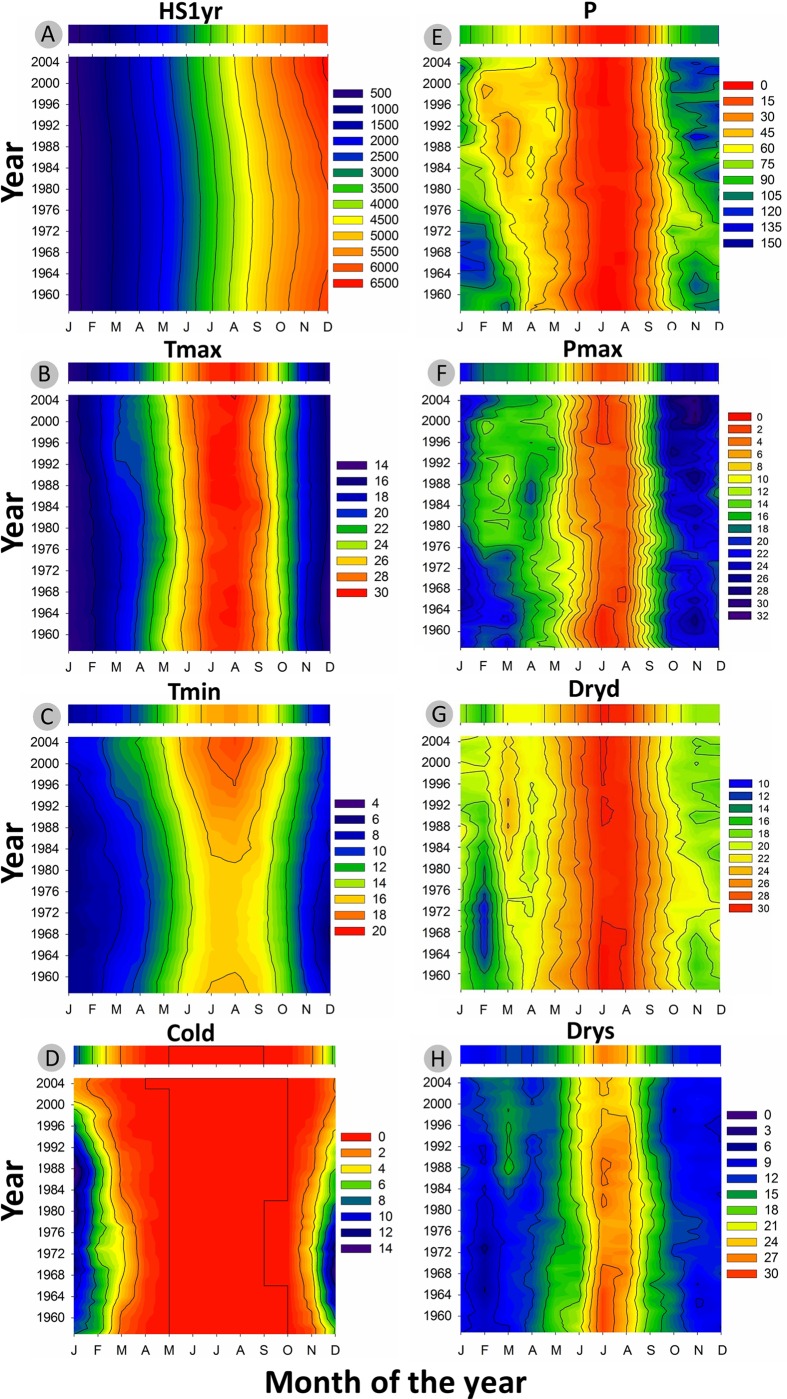
**2D representation of the recent evolution of monthly climatic variables (1958-2011) smoothed with a 15-year moving average window.** The colored bar above each graphic represents the long-term average over the period 1950-2012. **(A)** Heat sum of daily mean temperature from 1st of January in °C (HS1yr). **(B)** Monthly average of maximum temperature in °C (Tmax). **(C)** Monthly average of minimum temperature in °C (Tmin). **(D)** Number of cold days with Tmin <5°C (Cold). **(E)** Monthly accumulated precipitation in mm (P). **(F)** Monthly maximum precipitation (Pmax). **(G)** Maximum number of dry days (Dryd). **(H)** Maximum number of consecutive dry days (Drys).

The inter-annual evolution of climatic variables (Tmax, Tmin, Cold, P, Drys, PET, and SPEI) from 1950 to 2012 and according to Loess trends is presented in **Figure 3**. Monthly basis changes of temperature-based variables (HS1yr, Tmax, Tmin, Cold) and precipitation-based variables (P, Pmax, Dryd, Drys) are shown in **Figure 4**.

Minimum daily temperature (Tmin) was the most affected by recent climate evolution in our sampling location (**Figure 3B**). After Tmin significantly dropped from 1950 to 1972, the slope inverted between 1972 and 1975 and was followed by an increase of ∼3.5°C until 2012 (**Figure 3B**). Tmin showed the same behavior within each month of the monthly time-series, with larger amplitudes in fall and winter (**Figure 4C**). This led to the progressive decline of the number of cold days (Cold) since the 1980s, to reach the point of disappearing (**Figures 3C** and **4D**). Changes in Tmax were much less perceptible, except for a slight positive anomaly in spring since the 1990s (**Figure 4B**). There was only a slight increase of +0.5°C as compared to the average of the studied period. As a consequence, the mean temperature (T) showed an intermediate behavior between Tmin and Tmax, with an increase of ∼1.5°C with respect to the period average (not shown).

Precipitation did not show any significant trend over the study period (**Figure 3D**), fluctuating around an average annual amount of 683 mm. Nevertheless, a recent increase in the variability of precipitation expressed by a raising amplitude of fluctuations around the trend could be noticed between 1995 and 2010. After the extreme drought of 2005, there was a large increase in precipitation, which reflected in the SPEI index (**Figure 3G**).

Seasonally, there was a sharp decline of spring precipitation progressively spreading from spring to late winter from 1950 to 2012 (**Figure 4E**). The number of dry days (Dryd, Drysp) presented the expected opposite behavior as compared to precipitation (**Figures 4G,H**). The depletion of precipitation in spring resulted in the raise of the dry periods length mostly in March since the 1980s (**Figures 4E,G,H**). Over the same period the SPEI index showed decreasing values (**Figure 3G**) emphasizing the occurrence of more frequent droughts. These droughts were linked to the accentuated raise of temperature since the late 1970s inducing a higher evapotranspiration rate (PET) (**Figure 3F**) and driving the local water balance toward an unfavorable growing deficit. By contrast, precipitation tended to intensify in fall since the 1980s (**Figure 3D**). This was also reflected on maximum daily precipitation, which showed an increment in the months of October-November, concurrent with a clear drop in February and March during the same time lapse (**Figure 4F**).

### Discarded Climate Variables

The GDD and the heat sum since 1st of January (HS1yr) resulted in nearly identical correlation patterns when linked to wood xylogenesis. This also occurred with the maximum daily precipitation (Pmax) and the 95th precipitation percentile (P95). Therefore and to avoid redundant information we further discarded GDD and P95 variables in the rest of the manuscript.

### Effects of Recent Climate Changes on Wood Radial Growth

**FIGURE 5:**
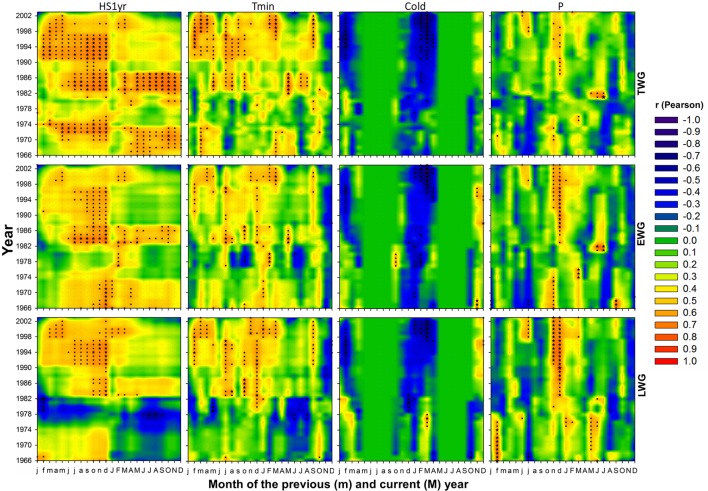
**Contour plot representation of 15-year monthly moving correlations of annual **(top)**, earlywood **(middle)**, and latewood **(bottom)** ring radial growth versus climate variables: cumulated heat sum over 1 year (HS1y), minimum temperature (Tmin), number of cold days with Tmin < 5°C (Cold) and precipitation > 0.1 mm (P), respectively from left to right.** Months of the current year are in upper case letters (M) while months of the year preceding the annual ring growth are in lower case letters (m). The stars are scaled according to the level of statistical significance of correlations (*n* = 15), with *p* < 0.05 for |*r* |≥ 0.515, *p* < 0.01 for |*r* |≥ 0.637 and *p* < 0.001 for |*r* |≥ 0.818, respectively.

**FIGURE 6:**
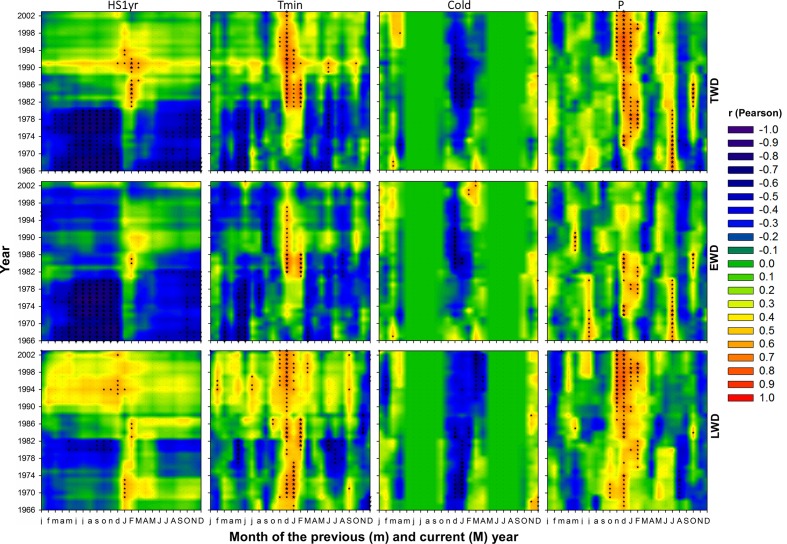
**As in **Figure 5**, but respecting to the effect of climate variables on tree-ring radial density**.

**FIGURE 7:**
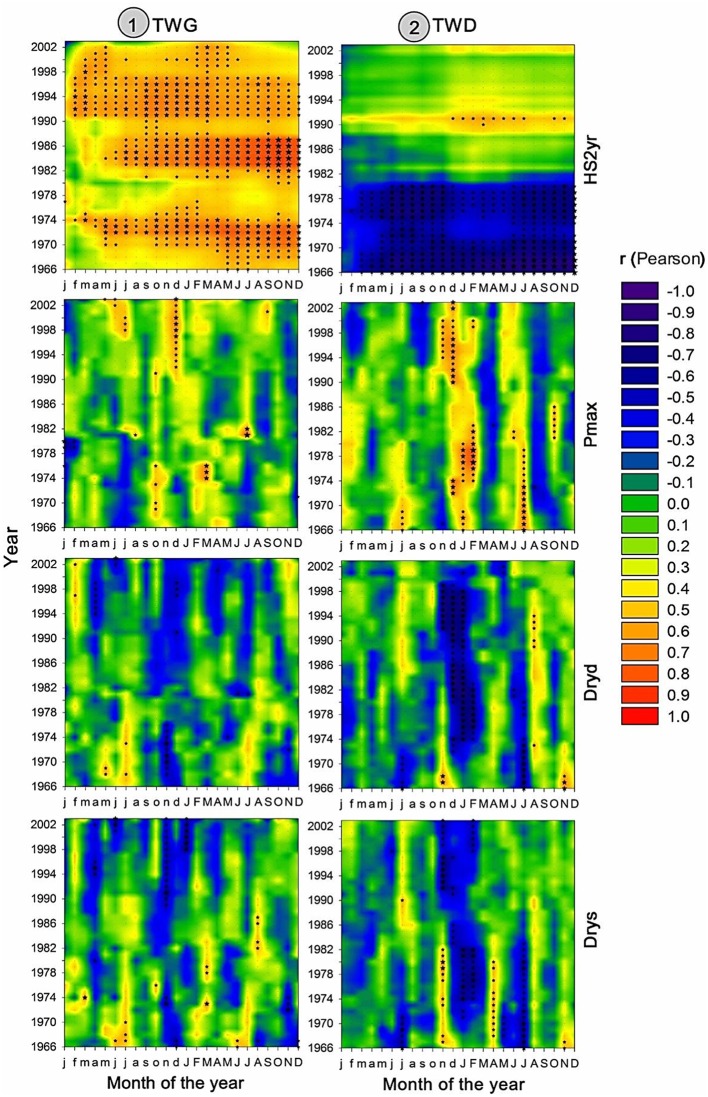
**As in **Figure 5**, but respecting to the effect of HS2yr, Pmax, Dryd, and Drys on TWG (left) and TWD (right)**.

**FIGURE 8:**
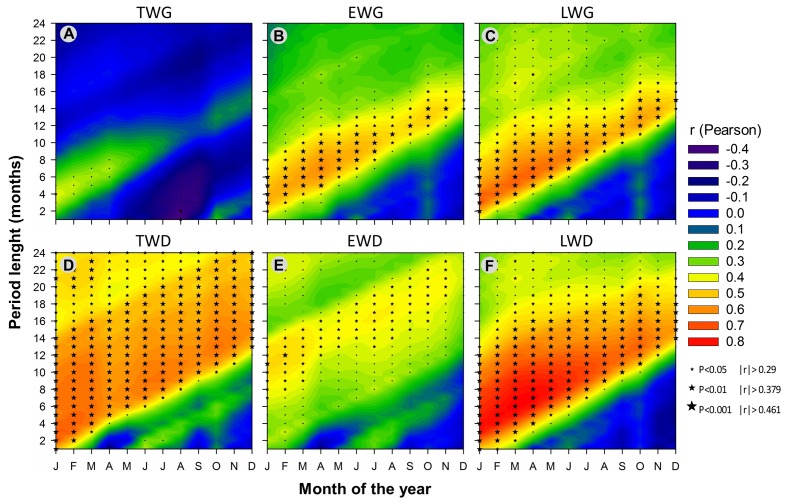
**Correlations between monthly time series of SPEI climate index and the corresponding standardized dendrochronological time-series over the period 1965-2010: total **(A)**, earlywood **(B),** and latewood **(C)** ring width; total **(D)**, earlywood **(E)**, and latewood **(F)** ring density.** Stars indicate significant correlations (*n* = 46). The stars are scaled according to the level of statistical significance with *p* < 0.05 for |*r*|≥ 0.29, *p* < 0.01 for |*r*|≥ 0.379 and *p* < 0.001 for |*r*|≥ 0.461, respectively, with *n* = 46.

**FIGURE 9:**
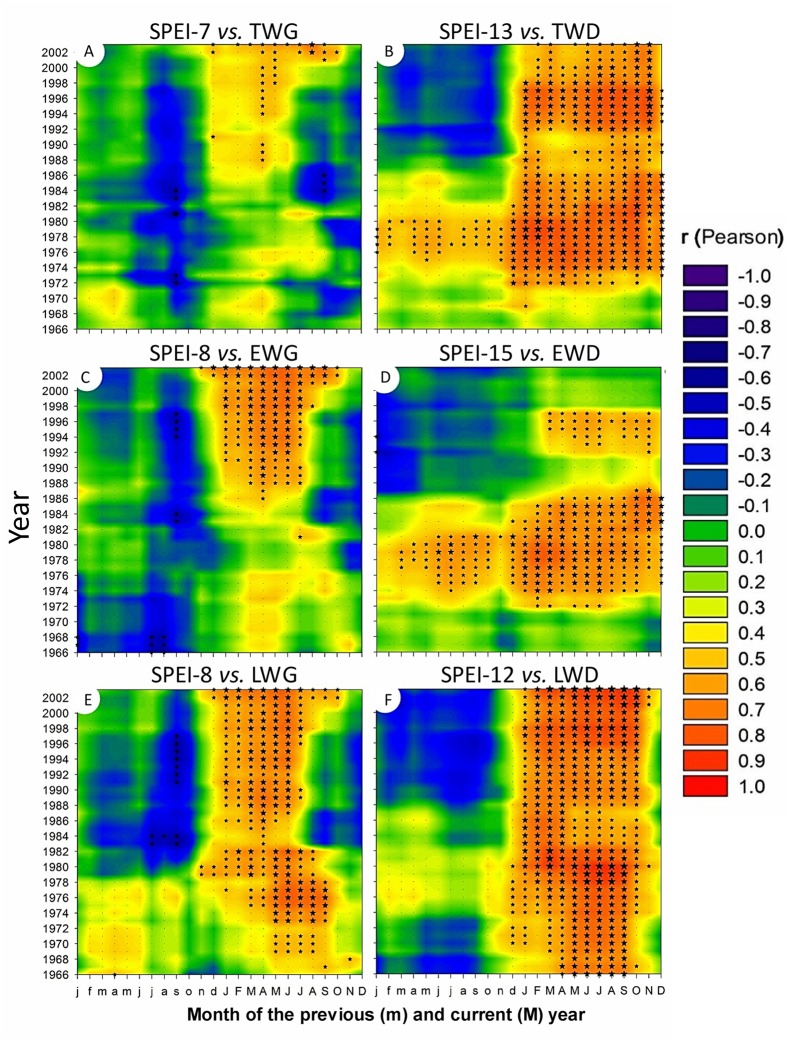
**Two dimensional representation of 15-yr monthly moving correlations of TWG and TWD versus SPEI drought index with the time scale providing the highest amount of significant correlations (cf **Figure 10**).** Total **(A)**, earlywood **(C)**, and latewood **(E)** ring width versus 7 to 8-Month SPEI index; Total **(B)**, earlywood **(D)**, and latewood **(F)** mean ring density versus 12 to 15-Month SPEI index.

**FIGURE 10:**
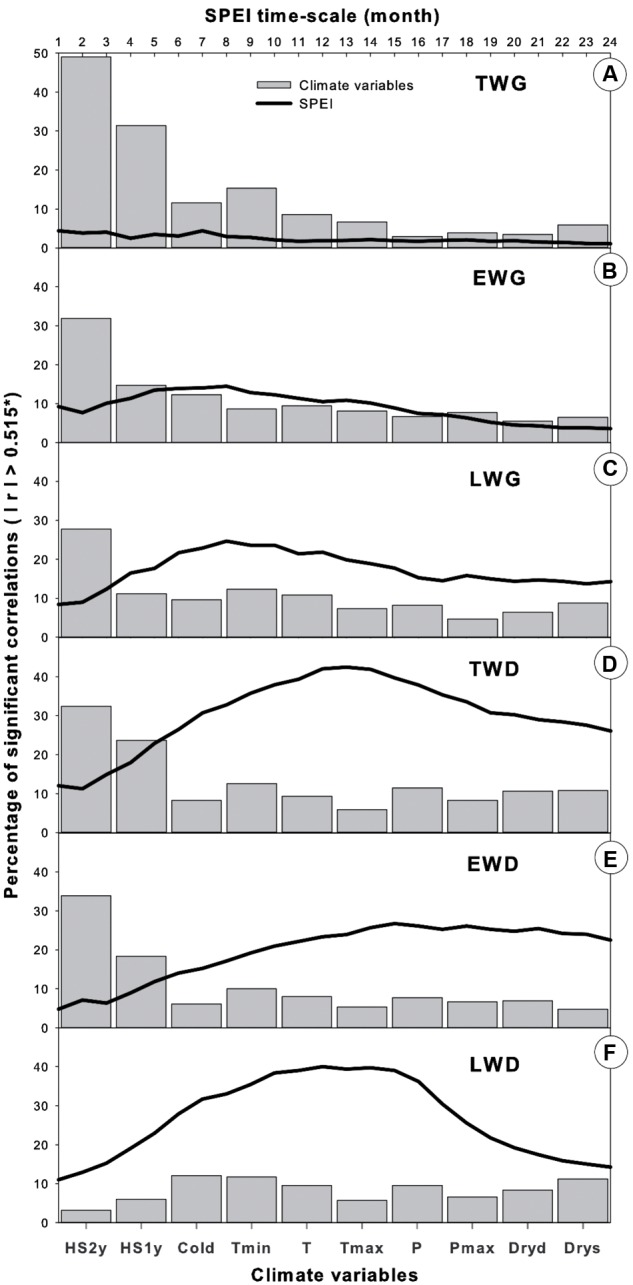
**Comparison of climate variable effects on *Pinus pinaster*’s dendrochronological traits, calculated as the percentage of significant correlations (*P* < 0.05) obtained over the total number of correlations calculated in each dataset (such as in **Figures 5**–**7** and **9**).** **(A)** TWG, **(B)** EWG, **(C)** LWG, **(D)** TWD, **(E)** EWD, **(F)** LWD.

Tree radial growth and density versus monthly climate variables relationships are, respectively, presented in **Figures 5**–**7**. **Figures 8** and **9** expose dendrochronology/SPEI relationships. The comparative effect of each climate variable is shown in **Figure 10**.

Tree radial growth was more sensitive to temperature than precipitation (**Figures 5** and **10**). TWG was mostly positively correlated with temperature and more strongly and frequently with Tmin (**Figures 5** and **10**). Significant correlations were stronger and more frequent with Tmin during most of the entire year preceding the ring formation. The temperature effect was cumulative, as reflected by the more solid and frequent correlations obtained with heat sums (HS1yr, HS2yr) of the year preceding the ring formation (**Figures 5** and **7**). The sign and level of correlation between TWG and Tmin (**Figure 5**) followed the evolution of Tmin trends since 1958, dropping from 1958 to ∼1982 and increasing again afterward (**Figure 3**). LWG response to temperature showed a more drastic alteration in ∼1982, shifting from slightly negative to positive correlations. Tree radial growth (TWG, EWG, and LWG) positively reacted to the disappearance of cold days (Cold) since 1986 with an increasing significance until 2012 (**Figure 5**).

Tree radial growth was consistently and positively correlated to winter precipitation and its intensity (**Figures 5** and **7**). Significant positive correlations with P and Pmax (and negative with Dryd and Drys), were mostly concentrated in November-January preceding the ring formation. TWG and LWG were the most sensitive to winter precipitation (**Figure 5**). TWG positive response to Pmax intensified since the 1990s with a shift from October to December, matching Pmax seasonal changes (**Figures 4** and **7**).

Tree radial growth was negatively affected by water deficit (**Figures 5**, **7**, and **10**). It showed hardly any significant correlation with SPEI (**Figure 8**). Nevertheless, significant relationships with SPEI accumulated over 7-8 months intensified since the late 1980s (**Figure 9**). LWG was the most affected by water deficit, while EWG showed an intermediate response to SPEI, as compared to TWG (**Figures 8** and **9**). TWG also negatively reacted to the reduction of rainy days in spring (Dryd, Drys) observed in the 1990s (**Figures 4** and **5**).

### Effects of Recent Climate Changes on Wood Radial Density

Maritime pine wood density severely responded to the inversion of temperature trend (**Figures 6** and **7**). Also the number of significant correlations and their magnitude were proportional to the amplitude of temperature change (**Figures 6** and **7**). Within temperature-based variables, TWD was more sensitive to heat sum and Tmin (**Figures 6** and **10**). There was a radical cleavage in the correlation pattern linking TWD to temperature-based variables (HS2yr, HS1yr) in ∼1980 (**Figure 6**). Before 1980, wood density was mostly negatively correlated to T, Tmin, and Tmax and essentially during the spring period of the previous and current year of the growing ring. After 1980, significant positive relationships became dominant in winter preceding ring xylogenesis (**Figure 6**) and appeared to be linked to the disappearance of cold days (**Figures 3**, **4**, and **6**). The relationships with cold days was more consistent for LWD (**Figure 6**). After ∼1997, correlations between EWD and temperature weakened (**Figure 6**) while T and Tmin stabilized during the same time lapse (**Figures 3** and **6**).

Positive correlations were observed between TWD and P-based variables in July of the current and previous years of wood xylogenesis, but only until ∼1975 (**Figure 6**). After ∼1972, significant positive correlations appeared during the winter period preceding ring growth (**Figure 6**). After 1972, at a more mature stage of the trees, there were strong and consistent positive (negative) relationships between TWD and winter P (winter Dryd-Drys) (**Figures 6** and **7**). EWD was the most sensitive to the lack of water availability during summer at the juvenile stage (**Figures 6** and **7**). LWD, however, tend to benefit from from the lack of summer precipitation before reaching 20 years old, yet it was more consistently linked to winter precipitation since the juvenile stage, as compared to EWD (**Figure 6**).

Interestingly, EWD was more sensitive to P-based variables during the coldest period (1972-1986) and increasingly less sensitive afterward despite the aggravation of water deficit (**Figures 6** and **9**). Latewood density showed the opposite pattern, demonstrating an increasing sensitivity to winter precipitation (**Figure 6**) as well as an increasing benefit of long-term water deficit until 2012 (SPEI-12, **Figure 9**). After the 1980s, latewood density was mostly controlled by winter T and P preceding the ring growth. The synergy between T and P effects on LWD seems to be linked to the strong relationships observed between LWD and the SPEI index (**Figures 6** and **9**) and intensifying since the 1980s.

Overall, TWD was more sensitive to SPEI-13, EWD to T-based variables and especially heat sum and LWD was more sensitive to SPEI accumulated over 12-15 months and P-based variables (**Figure 10**).

### Intra-Annual Density Fluctuations

**FIGURE 11:**
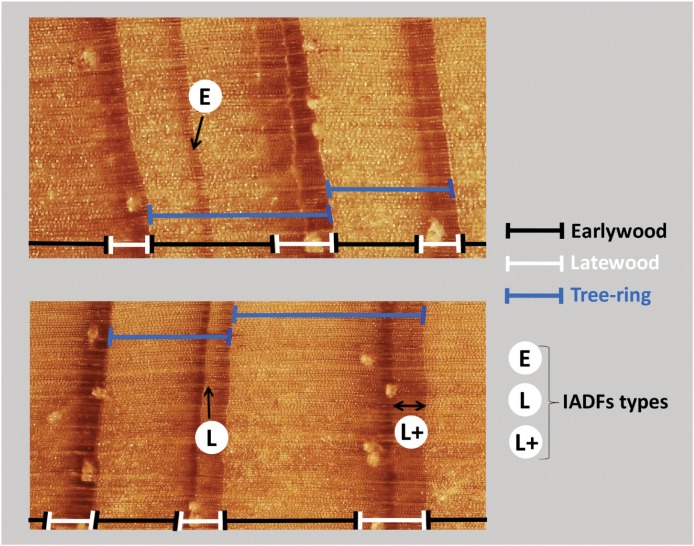
**Identification of IADFs within *P. pinaster* tree-rings.** IADFs were distinguished as latewood-like cells within earlywood (type E), as earlywood-like cells within latewood (type L) and as earlywood-like cells at the end of latewood and presenting a band with homogeneous color (type L+).

**FIGURE 12:**
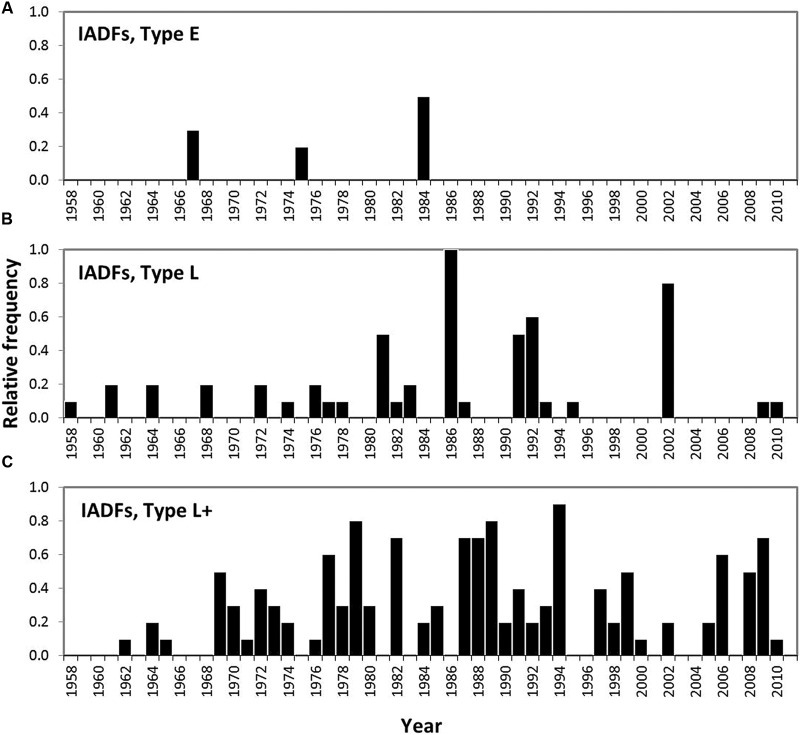
**Relative frequency of the occurrence of IADFs observed in 10 *P. pinaster* trees in southern Portugal.** IADFs type E **(A)**, IADFs type L **(B)**, IADFs type L+ **(C)**.

**FIGURE 13:**
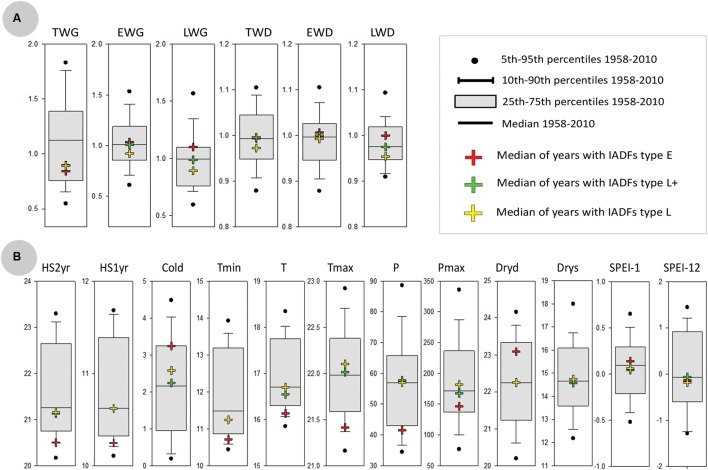
**Comparison of years with IADFs occurrence with all years of the period 1958-2011.** **(A)** Boxplots for the distribution of dendrochronological traits over the entire period 1958-2011, including the median, the interquartile range and upper and lower 5 and 10 percentiles. Also shown the dendrochronological traits corresponding to the years with IADFs occurrence (colored crosses). **(B)** As in **(A)**, but respecting to the distribution of annual climate variables (with units as in **Figure 5**).

The different types of IADFs E, L, and L+, distinguished in maritime pine cores are identified in **Figure 11**. The relative frequencies of their occurrence is presented in **Figure 12** and **Figure 13** shows a comparison of years with IADFs to all years covering the period 1958-2011.

IADFs located in earlywood (type E) were rarely observed in our samples (**Figure 12A**). They only accounted for 2% of the total number of tree-rings observed. IADFs type E occurred on years 1967, 1975, and 1995 (**Figure 12**) with particularly high LWG and LWD (percentiles 75 and 60, respectively) (**Figure 13A**). Those years were also exceptionally cold and dry (**Figure 13B**).

Intra-annual density fluctuations type L and L+, located in latewood, were much more frequent. They, respectively, accounted for 11 and 26% of the total number of tree-rings. IADFs type L occurred in years with low radial growth (TWG, EWG, and LWG), and years with low LWD (**Figure 13A**). They also tend to happen under colder conditions (**Figure 13B**) though not as cold as for IADFs type E.

Intra-annual density fluctuations type L+ were the most frequently observed. They occurred on 35 out of 53 years, in years with low radial growth (TWG), such as IADFs types L. However, they do not seem to be linked to climate variables, at least on an annual basis (**Figure 13B**). Taking into account the contrasting climatic conditions along mainland Portugal, the results related with IADFs highlighted the need of a detailed monthly basis analysis, which is nevertheless out of the scope of the present work.

## Discussion

### Recent Climate Evolution in Central Portugal

The recent evolution of climate variables in southern Portugal is in agreement with the recent Fifth Assessment Report of the IPCC (2013). The latter shows similar temperature increase trends at the global scale since 1958, with a decline until 1978 followed by an increasing trend since. This led to the disappearing of cold days in our sampling location. This trend was confirmed by the IPCC report describing the decline of cold days since 1950 and attributed to an anthropogenic cause with a very high likelihood.

While a slight annual precipitation decline has often been reported in mainland Portugal between 1951 and 2010 (Sousa et al., 2011; Santo et al., 2014b), our results, using the ECAD climatic dataset, do not present a clear evidence of such trend. Nevertheless, we noticed significant changes in the seasonal patterns of precipitation, namely a descent of precipitation conveyed by a raise of dryspell in spring and the intensification of precipitation in fall since the 1980s. Those changes have also been previously reported by Miranda et al. (2006) and more recently by Santo et al., 2014a,b).

The decline of SPEI index observed since the late 1980s is in agreement with the results of Vicente-Serrano et al. (2010) in Spain. It is most likely the result of the increasing evaporative demand due to the raise of temperature, since annual precipitation did not change significantly.

### *P. pinaster* Wood Growth Response to Recent Climate Changes

Consistent correlations patterns in our results showed that wood ring growth and density in maritime pine appeared to be more affected by temperature-based than precipitation-based variables when considering climate variables independently on a monthly basis (**Figure 10**). Wood radial increment and density both showed strong response to heat sum and Tmin (**Figures 5**–**7** and **10**), clearly following the changes of Tmin trends (**Figures 3**, **6**, and **7**). In addition, wood xylogenesis reacted very positively to the recent disappearance of cold days (**Figures 5** and **6**). The latter resulted in a larger number of days with Tmin above 5°C allowing cambium activity (Vieira et al., 2014), which led to the extension of the growing period. Overall our results expose the benefits of the increase of accumulated temperature and especially minimum temperature on *P. pinaster*’s wood dendrochronological traits, in particular since the 1980s.

Winter precipitation preceding the ring formation displayed consistent positive correlations with wood radial growth (**Figures 5** and **7**). These results agree with those of Vieira et al. (2009, 2010) who showed that a drier winter prior to growth had a negative impact on maritime pine tree ring width. Also *P. pinaster* wood growth seemed to be more sensitive to the seasonal changes of precipitation patterns rather than to annual fluctuations since 1958. The species seemed affected by the recent precipitation deficit in May of the year preceding the ring formation, mostly since the 1990s (**Figures 4**, **5**, and **7**) and though spring precipitation has been shown to decline since the 1960s (Paredes et al., 2006). The decline of spring precipitation might affect water storage, inducing precocious water deficit for the following year. Hence the relationships found between wood ring properties and SPEI drought index accumulated over several months identify the increasing long-term water deficit as a key-factor of the loss of productivity for *P. pinaster* in southern Portugal.

The response of *P. pinaster*’s wood radial increment to precipitation-based variables emphasize the importance of the storage of precipitation water from fall to winter (and even recently spring) preceding the growing period.

### *P. pinaster* Wood Density Response to Recent Climate Changes

According to Campelo et al. (2007), climate can explain up to 76% of the variability of pine ring density in Portugal. The changes in temperature trend observed in our work seems to affect severely the ring mean density, though with correlations showing less consistency and more complexity than those obtained with ring radial growth. Ring density was more affected by the temperature of the entire year before the 1980s, and mostly by winter temperature afterward.

Overall, EWD showed a less consistent and more puzzling response to climate variables than latewood. This is likely because earlywood is rather formed at the expense of stored carbohydrates than current photosynthesis (Hill et al., 1995). Besides, EWD undergoes a strong genetic control comparatively to latewood components (Nicholls et al., 1980; Zhang and Jiang, 1998). This is also true for maritime pine in Portugal (Louzada and Fonseca, 2002; Gaspar et al., 2008). In our study, EWD climatic response strongly changed from the juvenile to the mature stage. The juvenile phase of trees appears to be characterized by a high phenotypic variance (Louzada and Fonseca, 2002), in which the genetic potential is fully expressed between 7 and 10 years of cambial age (Gaspar et al., 2008). This can be related to a higher sensitivity of young trees to climate fluctuations and land establishment. Here EWD exhibited sensitivity to spring water stress, as previously reported by Nabais et al. (2014). Our results also agree with those of Campelo et al. (2007) studying *P. pinea* in Portugal. The authors showed that earlywood formation was mostly pre-determined at the beginning of the growing season. EWD correlation to spring precipitation intensified since the late 1990s, suggesting an increasing negative effect of the decline of spring precipitation. Yet this intensification could also be due to a raise of genetic heritability with tree age according to Gaspar et al. (2008). Although age-related effect on dendrochronological traits was minimized by standardization, it should be noted that the intensification of *P. pinaster*’s response to climate change can also be linked to a higher sensitivity of wood xylogenesis to climate factors through time (Zhang and Jiang, 1998; Aguiar et al., 2003; Dorado Liñán et al., 2012; Campelo et al., 2013, 2015; De Micco et al., 2016).

By contrast, LWD was more consistently and strongly correlated with climatic conditions than EWD. This is in agreement with Campelo et al. (2007), who stated that latewood development was more sensitive to climate variations compared to earlywood. Latewood formation mainly depends on current photosynthesis products, more closely controlled by current climate conditions (Zhang, 1997; Lebourgeois, 2000). We found that LWD was positively correlated with climatic conditions favoring wood growth. Propitious years with sufficient stored water at the beginning of the growing season tend to increase the length of the growing period and, therefore, the duration of tracheid maturation and carbon deposition, which in turn results in thicker cell walls and thus higher wood density (Wodzicki, 1971).

On the other hand, LWD was increasingly affected by long-term water deficit since the 1980s, negatively responding to the decline of SPEI index. Water deficit appears to affect the physiological processes involved in the allocation and utilization of carbohydrates stored toward the end or even after the growing season (Kozlowski and Pallardy, 1997). Nabais et al. (2014) suggested that the reduction of carbon assimilation due to lower precipitation in winter and spring might prevent the completion of cell wall deposition in latewood tracheids, leading to less dense earlywood-like cells. In addition, water stress has been shown to influence ring density fluctuations by a direct effect on cell volume affecting the lumen diameter and linked to the trade-off existing between hydraulic safety and hydraulic efficiency (Wilkinson et al., 2015). Besides restricting evapotranspiration and carbon assimilation through the regulation of stomatal conductance, water stress can also induce alterations in carbon allocation toward roots then becoming a priority carbon sink (Kurz-Besson et al., 2006). This would prevent further cell wall thickening in the trunk and reduce wood density.

Severe abiotic conditions during the growing season such as water stress may generate the production of latewood-like cells within earlywood or earlywood-like cells within latewood affecting the tree-ring density profile (Olivar et al., 2015). Our results show that false rings (IADFs) mostly occurred on years with low radial wood growth, pointing out non optimal, or stressful environmental conditions. IADFs in earlywood occurred very rarely in our location. They appear to be quite unusual in Mediterranean pine species (Battipaglia et al., 2016). Vieira et al. (2010) also reported rare earlywood IADFs in 100-yr old *P. pinaster* from western Portugal. This is attributed to the rarity of drought events during earlywood formation (Vieira et al., 2010; De Micco et al., 2016). We found that earlywood IADFs coincided with extreme cold and dry years. This is in agreement with our results on EWD showing stronger relationships with climate during the coldest years of the studied period (1972-1986). IADFs types L were much more frequent than IADFs_E. This was also reported by De Micco et al. (2016), who considered them as an extension of the wood formation promoted by a combination of summer drought and favorable conditions in late summer and early autumn. In our study L-IADFs appeared under less extreme cold climate conditions than type E. This is in contradiction with Campelo et al. (2013) who found that latewood false rings happened on years characterized by warm December. The effect of climate variables (considered on an annual basis) on IADFs type L+ was more elusive in our results, probably due to a higher effect of biotic factors such as tree heritability or aging, or abiotic factors such as nutrient availability or soil heterogeneity influencing root distribution and tree competition. Battipaglia et al. (2016) considered IADFs type L+ as transitional wood rather than true density fluctuations, after analyzing carbon and oxygen isotopic signals of the different kinds of fluctuations. On another hand, Vieira et al. (2010) and Campelo et al. (2013) showed that the IADFs type L+ were preferentially triggered by seasonal climate variables, namely favorable previous winter precipitation and favorable conditions before summer. The authors suggested that IADFs constitute a potential mechanism for individual trees to adapt to drought in *P. pinaster*, as they are mediated by stem size. On the other hand, tree radial growth and stem size is also strongly influenced by rooting depth, tree access to deep water sources during the dry years and groundwater fluctuations (Sun et al., 2000; Ford and Brooks, 2003)

### Groundwater Recharge as a Main Driver of *P. pinaster’s* Productivity and Quality

It is assumed that juvenile wood turns into mature wood 15–20 year from the pith (Fries and Ericsson, 2009). Our results agree with this last statement by showing a shift of significant correlations between climate and EWD (as well as LWD) from spring of the current year to winter of the previous year while changing from the juvenile to a more mature stage. In our study, the anatomical transition occurred around 1972, when trees were about 20 years of age (considering that 14 rings were formed between 1958 and 1972 and that trees needed 6 years to reach breast height where the tree-ring core were sampled).

We attribute those anatomical shifts to an alteration of tree water source from recent shallower water sources to older and deeper stored ones. This is in agreement with the study by Danjon et al. (2013) on *P. pinaster* in the South-West of France, showing the emergence of sinker roots on 5 years old trees and the expansion of deep roots after 12 years, becoming more abundant, thick and deep after 19 years. The authors also show that deep roots have a high overall tapering rate, especially during drought periods. This has also been shown for co-occurring ligneous species such as *P. halepensis* and *Quercus suber* (Filella and Peñuelas, 2003; David et al., 2013; Kurz-Besson et al., 2014). Maximum rooting depth reported for the three co-occurring species was lower than 7m depth (Canadell et al., 1996). According to Gómez and Viñas (2011), most of the *P. pinaster* root volume remained in the soil unsaturated zone, and only deep roots connected to the water table (wet season) or the capillary fringe (dry season). The latter used to show important seasonal fluctuations following the groundwater table depth (Ronen et al., 2000).

We showed that winter precipitation provides the largest amount of water in our location (**Supplementary Figure S3**). Since water availability in shallow soil horizons is not a limiting factor during winter and since the physiological activity of *P. pinaster* is minimum at this time of the year (Vieira et al., 2014), our results strongly implies that *P. pinaster*’s growth is favored by an optimum recharge of deep water sources during the rainy period. This statement is further strengthened by several studies performed in nearby locations of the Portuguese Alentejo region, which have shown that the aquifer recharge usually occurs 3 to 4 months after the onset of rainfall following the summer drought period (Francés, 2008; Ribeiro and Veiga da Cunha, 2010).

The fluctuation of aquifer recharge could also have been responsible for the stronger relationships observed between LWD and accumulated SPEI since the 1980s as the increase of temperature and PET most probably led to declining water storage in the region. Different studies predicting a significant decline of runoff process and river flow in the near future also predict a drop of the aquifer recharge in the southern Mediterranean basin within the near future (Fiseha et al., 2014; Mourato et al., 2015). For example, a decline of 40 to 68% of the annual groundwater recharge has also been predicted within 2050 in northern Morocco, associated to a drop of the groundwater level down to -5m depending on the heterogeneity of the region orography (Van Dijck et al., 2006). In South Portugal runoff is expected to fall 13 to 90% by the end of the century (Mourato et al., 2015). According to our results, such drastic changes of the ecosystem water balance are expected to induce dramatic consequences for *P. pinaster*’s wood productivity and quality for commercial purposes in the Alentejo region in the near future.

## Conclusion

In this manuscript, we analyzed and synthetized over 32000 correlations resuming the impact of the recent climate variability on dendrochronological traits of *P. pinaster* in southern Portugal between 1958 and 2011. Among the profusion of very interesting imbricated correlations exposed, we can highlight the two most striking ones:

(1) Our results underline a complex and antagonist response of wood formation to the combined effects of the recent temperature and precipitation changes. While thriving under the disappearing of cold days and the increase of minimum temperature, *P. pinaster* xylogenesis suffers from prolonged water deficit that has become more common.
(2) We show that *P. pinaster* trees in the Alentejo region of Portugal rely on groundwater sources to cope with Mediterranean droughts and are favored by better groundwater recharge at the end of the winter period.

Since the 1960s, southern Portugal has faced a significant increase of the aridity, drifting from a dry sub-humid to a semi-arid climate (Costa and Soares, 2012). Using best performing multi-model ensembles, Soares et al. (2015, 2016) predicted consistent rainfall reductions between 20 and 30% in this geographical area, according to the A1B and RCP8.5 greenhouse gas emission scenarios, for the end of the 21st century. With the same scenarios, Jacob et al. (2014) pointed out to an increase of the mean annual air temperature over 3°C in the southern Mediterranean area by 2100. Those predictions agree with the predicted collapse of multi-scalar drought indexes and the consequent aggravation of dryness in southern Europe by the end of the 21th century (Stagge et al., 2015). Such changes will likely exacerbate the aridification process, severely affect groundwater recharge and induce an acute drop of the capillary fringe and groundwater levels. According to our results, *P. pinaster’s* productivity and suitability for commercial purposes could be severely affected in the semi-arid area of the Alentejo region in southern Portugal.

Understanding how *P. pinaster* tree-ring traits are affected by climate changes under contrasting climatic conditions and according to the genetic variability of the species is still under assessment in Portugal. Outcomes of ongoing investigations will allow the characterization of the potential geographic distribution of maritime pine according to updated climate change scenarios. This will be crucial for plant breeders to select the most adapted proveniences and for policy makers and water managers to concept adaptation strategies to guaranty *P. pinaster*’s productivity and commercial benefits in Portugal in the near future.

## Author Contributions

CB’s achieved the calculation of meteorological indexes and the analysis of the climate evolution trends, the computation of dendrochronological/climate correlations, the interpretation and the redaction of the manuscript. IC provided the framework and part of the funding for the work to be undertaken. IC, JL, and MG all participated in the dendrochronological sampling. JL and MG performed dendrochronological measurements. TD participated in the conceptual design of the study. PS defined the meteorological datasets to be used for the study and the approach used for the meteorological analysis. RC assessed and shared the meteorological time-series. AR shared and adapted her Matlab algorithm for the computation of the SPEI multi-scalar drought index. FV shared and adapted the methodological approach used in a previous work. RT conceived the moving correlations methodology and supervised the team effort. CG provided complementary fundings for the work to be completed. She supervised the use of SPEI index in dendrochronological correlations and their related interpretation. CM performed a thorough detached analysis of the data interpretation, spotting unclear contents. Each of the co-authors performed a thorough revision of the manuscript, provided useful advices on the intellectual content and improved the English language.

## Conflict of Interest Statement

The authors declare that the research was conducted in the absence of any commercial or financial relationships that could be construed as a potential conflict of interest.

## Abbreviations

Cold: Number of days with minimum temperature <5°C
Dryd: Number of dry days per month
Drys: maximum number of consecutive dry days or Dryspell
EWD: earlywood mean density
EWG: Earlywood growth
HS1yr and HS2yr: Heat sum: daily mean T accumulated over 1 or 2 years
IADFs: intra-annual density fluctuations
LWD: latewood mean density
LWG: Latewood growth
P: monthly accumulated precipitation
PET: potential evapotranspiration
Pmax: maximum daily precipitation
T: monthly mean temperature
Tmax: Monthly average of maximum temperature
Tmin: monthly average of minimum temperature
TWD: ring mean density
TWG: Total annual ring width
