# Supplementary material for: Effects of Recent Minimum Temperature and Water Deficit Increases on Pinus pinaster Radial Growth and Wood Density in Southern Portugal
Source: Front Plant Sci. 2016 Aug 12;7:1170. doi: 10.3389/fpls.2016.01170 (PMC4982320; doi:10.3389/fpls.2016.01170)
Supplement: Supplementary file 1 [file Presentation_1.PDF]

Supplementary Material

The effects of minimum temperature and water deficit increases on *Pinus pinaster* wood radial growth and density in southern Portugal.

Cathy Kurz-Besson*, José L. Lousada, Maria J. Gaspar, Isabel Correia, Teresa Soares David, Pedro M. M. Soares, Rita M. Cardoso, Ana Russo, Filipa Varino, Catherine Mériaux, Ricardo M. Trigo & Célia M. Gouveia.

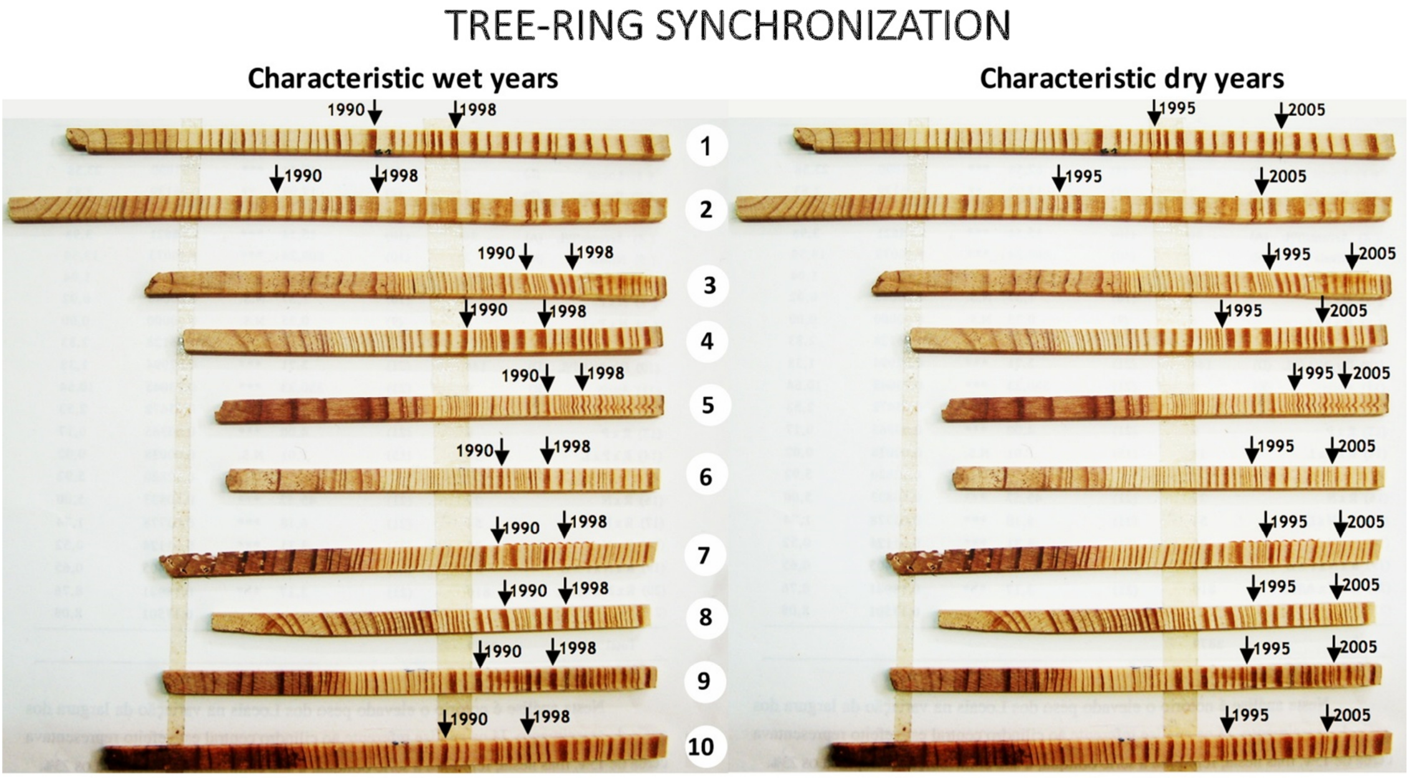

Supplementary Figure I. Synchronization of tree ring dating of the ten *P. pinaster* cores sampled in Companhia das Lezirias by reference to characteristic wet (1990, 1998) and dry (1995, 2005) years.

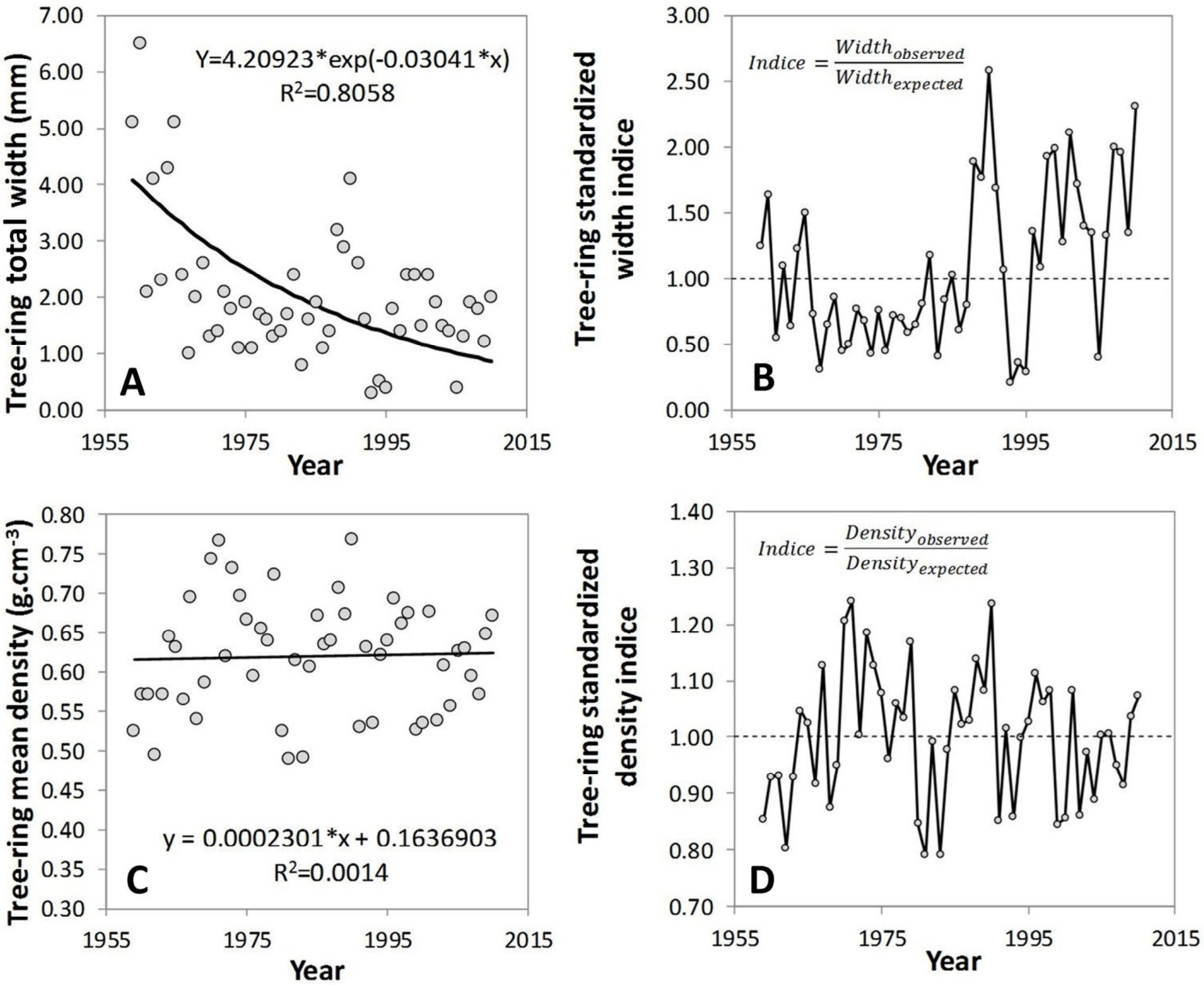

Supplementary Figure II. Example of the standardization performed on each sampled core to minimize tree age effect on dendrochronological traits. A. Exponential decay function applied on the time-series of tree-ring total width (TWG) obtained for *P. pinaster* tree nº7. B. TWG Standardized index calculated for tree nº7 by dividing the observed TWG by the expected TWG values of the fitted exponential function. C. Linear function applied on the time-series of tree-ring mean density (TWD) obtained for *P. pinaster* tree nº7. D. TWG Standardized index calculated for tree nº7 by dividing the observed TWD by the expected TWD values of the fitted exponential function.

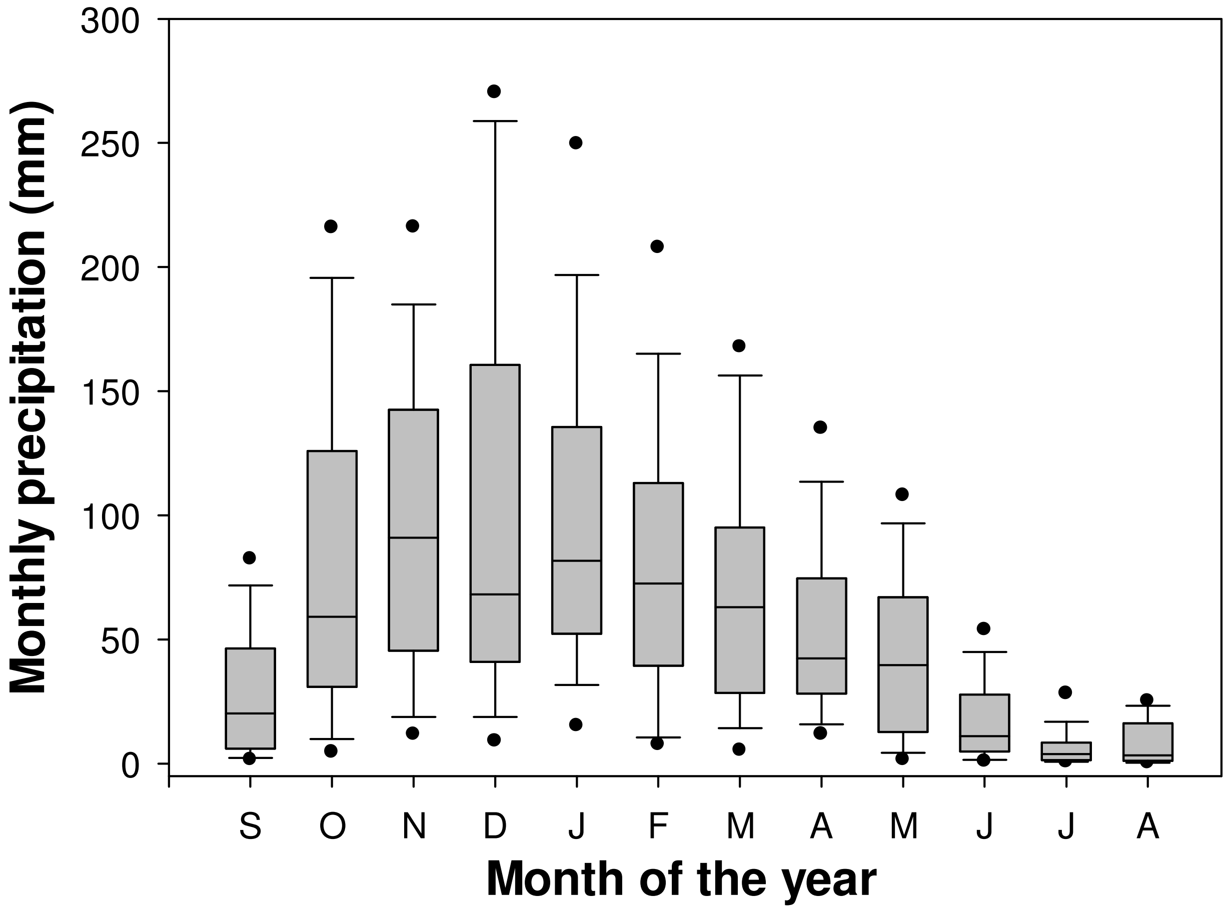

Supplementary Figure III.
Box plot representing the distribution of monthly precipitation over the period 1958-2011.
